# Leveraging advanced graph neural networks for the enhanced classification of post anesthesia states to aid surgical procedures

**DOI:** 10.1371/journal.pone.0320299

**Published:** 2025-04-25

**Authors:** Dongge Niu, Renxin Ru, Jiasheng Zhang, Yibo Zhang, Cheng Ding, Yao Lan

**Affiliations:** 1 Department of Anesthesiology, Peking University International Hospital, Beijing, China; 2 The Third Hospital Of Nanchang, NanChang, JiangXi, China; 3 School of international business, Anhui International Studies University, Wuhu, Anhui, China; 4 Nanomega CryoA.I. Corp., Beijing, China; 5 Gezhi Future Research Institute, Beijing, China; 6 School of Systems and Computing, UNSW, Kensington, Australia; 7 UNSW Canberra, ACT, Canberra, Australia; 8 Department of Biomedical Engineering, Georgia Institution of Technology, Atlanta, Georgia, United States of America; Universita degli Studi di Messina, ITALY

## Abstract

Anesthesia plays a pivotal role in modern surgery by facilitating controlled states of unconsciousness. Precise control is crucial for safe and pain-free surgeries. Monitoring anesthesia depth accurately is essential to guide anesthesiologists, optimize drug usage, and mitigate postoperative complications. This study focuses on enhancing the classification performance of anesthesia-induced transitions between wakefulness and deep sleep into eight classes by leveraging advanced graph neural network (GNN). The research combines seven datasets into a single dataset comprising 290 samples and investigates key brain regions, to develop a robust classification framework. Initially, the dataset is augmented using the Synthetic Minority Over-sampling Technique (SMOTE) to expand the sample size to 1197. A graph-based approach is employed to get the intricate relationships between features, constructing a graph dataset with 1197 nodes and 714,610 edges, where nodes represent data samples and edges are the connections between the nodes. The connection (edge weight) is calculated using Spearman correlation coefficient matrix. An optimized GNN model is developed through an ablation study of eight hyperparameters, achieving an accuracy of 92.8%. The model’s performance is further evaluated against one-dimensional (1D) CNN, and six machine learning models, demonstrating superior classification capabilities for small and imbalanced datasets. Additionally, we evaluated the proposed model on six different anesthesia datasets, observing no decline in performance. This work advances the understanding and classification of anesthesia states, providing a valuable tool for improved anesthesia management.

## 1 Introduction

The human brain is an extremely complex organ that controls states of awareness and rest through sophisticated neural processes. Understanding the neural mechanisms that regulate wakefulness and deep sleep, especially after anesthesia, is a critical area of study in neuroscience and clinical medicine. Anesthesia-induced transitions between states of consciousness that offer valuable insights into the brain’s fundamental functions. During periods of anesthesia, the brain’s normal activity is altered significantly, leading to transitions between wakefulness and deep sleep states. Accurate classification of these states is essential for optimizing post-anesthesia recovery and ensuring patient safety [1]. Traditionally, the monitoring of consciousness levels during anesthesia relies heavily on physiological metrics such as heart rate, blood pressure, and respiratory rate. However, these indicators can be inconsistent due to individual variability and are often insufficient for accurately determining the patient’s state of awareness [2,3]. This limitation underscores the need for reliable methods to assess brain states, especially following anesthesia, where the risk of complications due to improper anesthesia levels is significant [4,5].

Recent advancements in artificial intelligence (AI) and machine learning (ML) present a promising solution to this challenge. AI, encompassing a range of computational techniques designed to mimic human cognition, offers the potential to transform how we analyze and interpret complex datasets, such as those generated by neuroimaging [6]. These methods can simulate the brain’s neural processes, providing a nuanced understanding of different states of consciousness [1,7,8].

Unlike traditional approaches that rely on indirect physiological measures, AI-driven models can determine the patient’s state of consciousness with greater precision [9,10]. Traditional methods of monitoring and analyzing brain states often involve manual interpretation and lengthy procedures, which can be time-consuming and prone to error. In contrast, AI-driven systems can automate these processes, reducing the time required for diagnosis and minimizing human error [6,7]. Accurate classification of wakefulness and deep sleep states can help in tailoring recovery protocols to individual needs, thereby reducing the risk of complications such as postoperative delirium or cognitive dysfunction [4]. For clinical experts, the application of AI and ML in classifying brain states post-anesthesia provides a powerful tool for improving patient outcomes.

This study aims to classify the wakefulness and deep sleep states of post-anesthesia states utilizing the Graph Neural Network (GNN) model. For the classification, seven datasets are collected and merged, resulting in eight distinct classes. However, the combined dataset had a limited size of 290 samples. To address this, the dataset is augmented using the Synthetic Minority Over-sampling Technique (SMOTE) [11], increasing the total to 1197 samples. This augmentation is performed to enhance the dataset size for the classification task. Notably, the issue of data imbalance is intentionally left unaddressed. This decision is based on the fact that in real-world scenarios, collecting balanced data for each class is often challenging and time-consuming. Therefore, developing a model that can effectively handle imbalanced and small datasets is valuable for classifying anesthesia states. Despite the augmentation, the dataset size remains relatively small for training deep learning and machine learning models. Additionally, data imbalance poses challenges, as deep learning and machine learning models can potentially overfit classes with more data and underfit those with less data. To address the challenge, the GNN model is employed to accurately classify wakefulness and deep sleep states, constructing a graph dataset. This approach allows us to model the complex relationships and interactions between different brain regions, potentially leading to more precise and reliable classifications. This graph-based method emphasizes structural feature relationships and interactions, providing a detailed topological view that maintains strong connections. By enabling an in-depth analysis of these relationships, it enhances the understanding of feature-class interactions, particularly benefiting classification algorithms for small and imbalanced datasets [12]. For the graph dataset, initially a feature matrix comprising 1197 rows and 8 columns is generated, including six feature columns, a target column, and a unique identifier for each row. The relationships among the rows are explored by constructing a graph, where each row is considered as a node. The Spearman correlation coefficient is used to determine the edges between nodes. The resulting graph consisted of 714,610 edges and 1197 nodes. An ablation study is conducted on eight different hyper-parameters, leading to the development of an optimized GNN model that reached a maximum accuracy of 92.8%. The outcome is evaluated using multiple performance metrics and statistical analyses. Additionally, the performance of the proposed model is compared with one-dimensional (1D) CNN and six machine learning models. Furthermore, the proposed model was tested on six additional anesthesia datasets, confirming its consistent performance without any degradation. The major contributions of this study are,

- A dataset for classifying anesthesia-induced states of consciousness is generated by merging seven public datasets and augmented utilizing SMOTE.
- Compared the performance of GNN model utilizing SMOTE and three other methods to highlight the effectiveness of the proposed methodology.
- A graph dataset is generated, and a GNN model is proposed for the classification which demonstrated its effectiveness in handling imbalanced dataset.
- To achieve the optimal model configuration, the hyper-parameter tuning of the GNN model is conducted through an ablation study.
- Compared the GNN model’s performance with ten other machine learning models and ensemble models to present its efficacy.

## 2 Related works

Numerous studies have been conducted on depth of anesthesia (DoA) detection and classification using various approaches, including electroencephalography (EEG), functional magnetic resonance imaging (fMRI), and other neuroimaging and neurophysiological techniques. Meghna Roy Chowdhury et al., [13] introduced a cost-effective and advanced method for predicting DoA with deep learning models applied to 512 Hz electrocardiogram (ECG) and 128 Hz photoplethysmography (PPG) data. This study, conducted on signals from 50 patients at National Taiwan University Hospital (NTUH), achieved a maximum accuracy of 86% using a 10-layer CNN with ECG and PPG heatmaps as inputs. The method is particularly suitable for small hospitals due to its low-cost signals, minimal data reconstruction, and limited memory and timing constraints.

Sara Afshar et al., [3] developed a unique deep learning architecture incorporating an attention layer, bidirectional long short-term memory (LSTM), and convolutional neural networks inspired by the inception module. Their model outperformed existing DoA estimation methods, achieving a root mean square error of 5.59 ± 1.04 and mean absolute error of 4.3 ± 0.87, with an average 15% improvement in AUC. Nooshin Bahador et al., [14] explored the use of fused EEG-ECG data for tracking transitions between anesthesia stages through time-frequency ridge mapping, yielding 94.14% precision and a prediction time of 0.28 seconds, outperforming common data-level fusion techniques. Wala Saadeh et al., [15] developed a machine learning-based DoA classification processor using a 256-point fast Fourier transform accelerator to extract features like FBSE, SEF, and beta ratio. Tested on EEG data from 75 surgical patients, this processor, designed with 65 nm CMOS technology, achieved 92.2% average accuracy with a latency of 1 second and a classification energy consumption of 140 nJ. Jianfei Li et al. [16] introduced a novel methodology for multiscale feature extraction on graph structures through the development of Haar-type graph framelets that possess essential attributes, including permutation equivariance, computational efficiency, and sparsity. These framelets facilitate the proficient representation of hierarchical information within graph-structured data, which is vital for the accurate representation of both local and global features of graphs. Another comprehensive exploration is seen for advanced deep learning techniques for graph-structured data [17], offering theoretical insights, model developments, and practical applications that significantly contribute to the evolving field of graph neural networks. In another study, Li et al. [18] introduce a novel framework for bootstrapped graph representation learning, termed BLoG, which incorporates both local and global regularization mechanisms for the purpose of enhancing recommendation systems. Specifically, BLoG generates pairs of positive and negative instances by leveraging aggregated node features, drawing on two distinct perspectives of the original user-item graph architecture. In her EduCross, an advanced framework [19] she aimed at enhancing Cross-Modal Retrieval (CMR) in the context of multimodal educational slides, a field where traditional retrieval systems frequently exhibit suboptimal performance.

Ronglin L et al., [20] proposed a method combining EEG hybrid features with a sparse denoising autoencoder (SDAE) and LSTM, demonstrating superior performance over LSTM and other conventional indices. Muhammad Ibrahim Dutt et al., [21] presented a method using fractal features and the Stationary Wavelet Transform (SWT) with a multilayer perceptron (MLP) classifier, achieving a top accuracy of 96.8% and setting a new benchmark for DoA estimation. Sara Afshar et al., [3] also demonstrated a deep learning approach that continuously predicted BIS scores from two EEG channels on the forehead, outperforming traditional models in error rates and generalization. Qihang Wang et al., [22] introduced Anes-MetaNet, a meta-learning architecture for classifying brain states during anesthesia, proving effective in tests with office-based anesthetic EEG datasets. Similarly, Meng Shi et al., [7] designed Anes-MetaNet and reported high Spearman’s rank correlation (0.9344) between predicted and actual PSI values. Yue Gu et al., [23] evaluated DoA using multiple EEG variables combined with artificial neural networks (ANN), with accuracy rates of 84.4% for general anesthesia, 73.6% for mild anesthesia, 14% for deep anesthesia, and 86.4% for awake states, showing a significant correlation (0.892, p < 0.001) with BIS. Finally, Oscar Mosquera Dussan et al., [24] conducted an observational single-center study, finding a strong Pearson correlation between entropy module indices and the Complexity Brainwave Index during general anesthesia.

GNNs have gained significant attention for their ability to capture complex relationships in structured data, making them valuable for various classification tasks. In this context, Mahsa Mesgaran et al. [25] introduced an anisotropic graph convolutional network to mitigate over-smoothing, enhancing node classification accuracy. Their model outperformed baseline methods on citation networks and image datasets, demonstrating superior predictive performance. Similarly, Tanvir Hassan et al. [26] proposed RS-Net, a graph-based model for 3D human pose estimation that captures long-range dependencies using matrix splitting and adjacency modulation. Their approach achieved state-of-the-art accuracy on benchmark datasets, surpassing previous models. Furthermore, Ibrahim Salim et al. [27] developed a graph-based disease prediction model integrating imaging and non-imaging features, achieving 50% and 13.56% accuracy improvements on ABIDE and ADNI datasets, respectively. Their method significantly outperformed conventional GCN-based approaches for autism spectrum disorder and Alzheimer’s disease classification. Collectively, these studies highlight the growing impact of GNNs in uncovering intricate patterns across diverse domains.

There are still a few limitations in place despite the advances in depth of anesthesia (DoA) detection and classification. Numerous researches have a strong reliance on datasets, which could not translate well to more general applications. Furthermore, the high accuracy rates frequently result in complicated and computationally demanding models, which limits their usefulness in environments with limited resources. Moreover, obtaining predictions that are generally dependable is difficult due to individual differences in the physiological reactions to anesthesia. We present an enhanced way to detect the DoA in eight unique states using a graph neural network, improving the generalizability and reliability of the detection process across different clinical situations. Our approach achieves robust accuracy across diverse and mixed datasets.

## 3 Dataset description

To detect the effects after anesthesia, seven datasets [28] were collected in the study, including: Sleep dataset, Anesthesia dataset, eyes-open/eyes-closed (EO/EC) dataset, two unresponsive wakefulness syndrome (UWS) dataset, REM sleep dataset, and N3 sleep/UWS dataset. This study did not involve the direct recruitment of human participants. Instead, we used publicly available, fully anonymized data from the Global Signal and Random Time Points Database (https://zenodo.org/records/8348272). As the dataset does not include identifiable information, and no intervention or interaction with individuals occurred, the need for participant consent was not applicable. Furthermore, as the data were collected independently of this study and are openly available, no additional ethical review or consent process was required.

The activation of brain regions during the peaks of global signal and random time points were extracted under different arousal conditions. These datasets have the same attributes VPL, PUL, MD, WM, CSF, rVPL. After combining them we get eight classes which is Wakefulness (W), Drowsy Sleep (DS), Unresponsive Wakefulness Syndrome (UWS), Rapid Eye Movement Sleep (REM), NREM Stage 2 Sleep (N2), and the impact of Brain Injury (BI). A small explanation of these attributes in medical domain is provided below in Table 1.

**Table 1:** A small explanation of the features in medical domain.

Feature	Location and Function	Clinical Relevance
VPL (Ventral Posterior Lateral nucleus)	The VPL is located in the Thalamus,s which acts as a relay station for sensory and motor signals. The VPL specifically relays somatosensory information (touch, temperature, pain, and proprioception) from the body to the primary somatosensory cortex in the brain.	Damage or dysfunction in the VPL can lead to sensory deficits, such as loss of sensation or abnormal sensations (paresthesia) in the body.
PUL (Pulvinar)	Damage or dysfunction in the VPL can lead to sensory deficits, such as loss of sensation or abnormal sensations (paresthesia) in the body.	Abnormalities in the pulvinar can affect visual perception and attention. It is also implicated in certain neurological conditions such as visual neglect and some psychiatric disorders.
MD (Mediodorsal nucleus)	The MD nucleus is situated in the medial part of the Thalamus. It has connections with the prefrontal cortex and is involved in higher-order cognitive functions, including memory, executive function, and emotional regulation.	Damage to the MD nucleus can result in cognitive and memory impairments, often seen in conditions such as Korsakoff syndrome and schizophrenia.
WM (White Matter)	White matter consists of myelinated axons that form the communication pathways between different brain regions. It is essential for the efficient transmission of electrical signals throughout the nervous system.	White matter integrity is crucial for normal brain function. Diseases such as multiple sclerosis, leukodystrophies, and traumatic brain injury can affect white matter, leading to a wide range of neurological deficits
CSF (Cerebrospinal Fluid):	CSF is a clear, colorless fluid that surrounds and cushions the brain and spinal cord. It is produced in the ventricles of the brain and circulates through the subarachnoid space. CSF helps to protect the central nervous system, remove waste products, and maintain intracranial pressure.	Abnormalities in CSF flow or composition can indicate various neurological conditions, such as hydrocephalus, meningitis, and intracranial hemorrhage.
rVPL (Right Ventral Posterior Lateral nucleus)	The rVPL is the right-sided counterpart of the VPL nucleus. It has similar functions in relaying somatosensory information from the contralateral (left) side of the body to the sensory cortex.	Lesions or abnormalities in the rVPL can lead to sensory disturbances or loss of sensation on the left side of the body, impacting the patient’s ability to perceive touch, temperature, and pain accurately.

**Fig 1:**
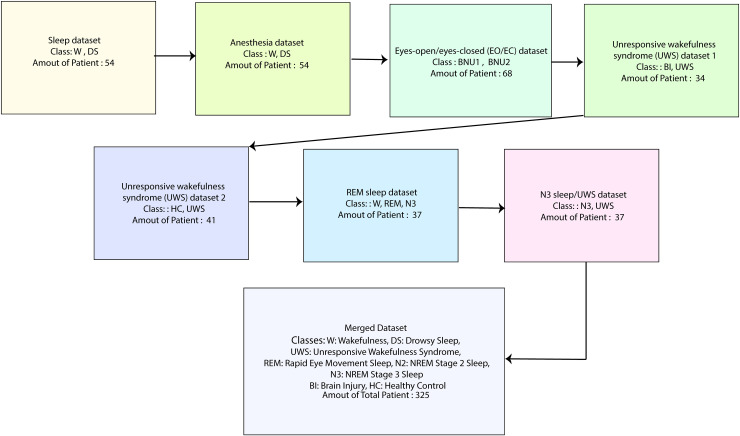
Illustration of the process of merging seven datasets with 325 patients.

These regions and structures play critical roles in sensory processing, cognition, and maintaining the overall brain. A visual representation of these datasets is shown in Fig 1.

## 4 Methodology

**Fig 2:**
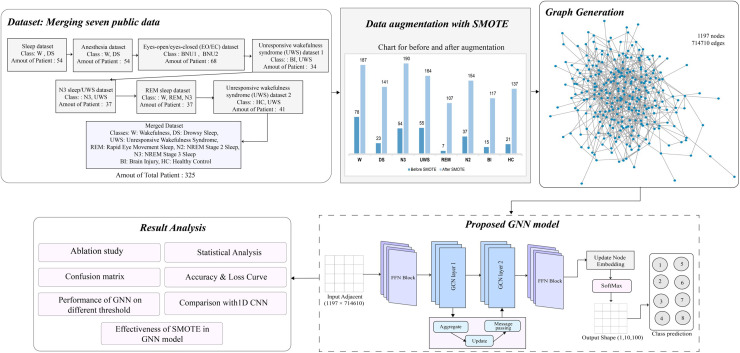
Proposed methodology.

This study presents an automatic approach for the identification of wakefulness and deep sleep states after anesthesia utilizing the GNN model. The proposed methodology of this study is demonstrated in Fig 2

As depicted in Fig 2, seven publicly available datasets are collected and merged for the classification task, resulting in eight classes. This combined dataset is highly imbalanced, with only 291 instances. To address this, the SMOTE augmentation technique is applied, increasing the number of instances for each class. Initially, the classes W, DS, N3, UWS, REM, N2, BI, and HC comprise 78, 23, 54, 55, 7, 37, 15, and 21 instances, respectively. After augmentation, these numbers increase to 187, 141, 190, 164, 107, 154, 117, and 137, respectively. Using the augmented dataset, a graph dataset is generated, where each row represents a node, and edges are created between nodes based on their Spearman correlation. This graph consists of 1,197 nodes and 714,610 edges. An ablation study is conducted to optimize the GNN model, involving hyper-parameter tuning for eight variables. The optimized GNN demonstrates superior performance in the classification task. The GNN model’s performance is further assessed with several statistical analyses utilizing the confusion matrix. The performance of GNN model is assessed with several threshold values (i.e., an edge is generated if the correlation value is equal to or below the threshold) of  ≤  0.7,  ≤  0.8,  ≤  0.9,  ≤  0.93,  ≤  0.97, and  ≤  1, where for  ≤  1 threshold which means with all the edges, the GNN model’s performance is highest. Additionally, the model’s performance before and after applying SMOTE is compared to highlight the effectiveness of the augmentation process. Finally, a comparative analysis is conducted between the proposed GNN and 1D CNN. The results show that the GNN model is the most effective among the three.

## 5 Graph generation process

The process of graph dataset generation is demonstrated in this section. Let us consider a graph $G=\left(V,E\right)$ that is composed of a set of nodes V and edges *E*. In this context, each node $v_{i}\in V$ represents an individual entity, and each edge $e_{ij}=\left(v_{i},v_{j}\right)\in E$ signifies a connection between two nodes $v_{i},v_{j}$. The neighborhood of a node $v_{i}$ is defined as $N\left(v_{i}\right)=\{m\in V|\left(v_{i},m\right)\in E\}$, where m is a neighboring node of $v_{i}$. An adjacency matrix *A* of size M×M is constructed where $A_{ij}=1$ if $e_{ij}\in E$ and $A_{ij}=0$ if $e_{ij}\in E$.

To generate the graph dataset, a feature table is first established, consisting of 1197 rows and 8 columns. These columns include six distinct features, a unique identifier for each row, and the target class label. Each row in this table is treated as an individual node *v* in the graph. The edge table is created by calculating the Spearman correlation coefficient between each pair of nodes, which quantifies the relationships between the features. The calculation involves determining the coefficient for each pair of rows to find the connection strength between the nodes. The formula used to calculate the Spearman correlation coefficient is given in Eq. 1 [29].


$$S=1-\frac{6\sum d_{n}^{2}}{p\left(p^{2}-1\right)}$$ (1)

where $d_{n}$ represents the differences between the ranks of the nodes, and *p* is the number of nodes. Table 2 shows the graph dataset, where target and source column represent the connected nodes and correlation column represents the weights of the edges.

**Table 2:** The generated graph dataset.

Target	Source	Correlation
1	2	0.734895
1	3	0.574287
1	4	0.717434
1	5	0.643757
1	6	0.61044
...	...	...
1193	1195	0.995192
1193	1196	1
1194	1195	0.99112
1194	1196	0.995706
1195	1196	0.995192

**Fig 3:**
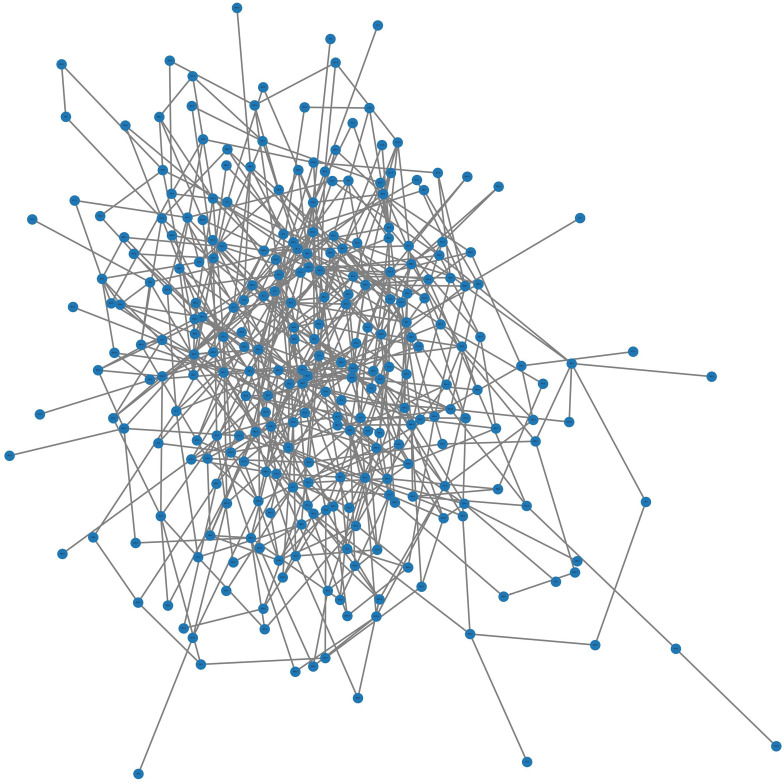
Graph visualization with 300 nodes and 600 edges.

The strength of the connection between two nodes is determined by the Spearman correlation coefficient, which ranges from -1 (perfect negative correlation) to 1 (perfect positive correlation), with 0 indicating no correlation. As the graph contains large number of nodes and edges, the full graph visualization becomes too dense to understand the nodes and their connections. However, a graph portion of the whole graph is randomly selected utilizing 300 nodes and 600 edges between the nodes for a graph visualization. Fig 3 shows the graph portion.

## 6 Model

GNNs are a specialized type of deep neural network tailored for processing graph-structured data. They excel in capturing the relationships and dependencies between nodes by leveraging the graph’s inherent connectivity. These models operate on data represented by nodes, edges, and edge weights, which serve as the primary inputs. By aggregating and transforming information from neighboring nodes, GNNs effectively learn and capture the embedded information from a node’s neighborhood within the graph structure. This section presents the proposed GNN architecture.

### 6.1 Feed-forward network (FFN) block

The FFN block in the proposed GNN model is meticulously designed to enhance performance and prevent overfitting issue. Each FFN block consists of three essential layers. The first layer is Batch Normalization, which normalizes the input data to stabilize and accelerate the training process by ensuring consistent distribution of features across different batches. This is followed by a dropout layer with a dropout rate of 0.3, which mitigates overfitting by randomly dropping out a fraction of neurons during training. Finally, the dense layer with the Rectified Linear Unit (ReLU) activation function is employed. It is a widely used activation function that introduces non-linearity while allowing for efficient gradient propagation during training, which is especially useful for smaller datasets. Let the ReLU as *σ* and input as *x*, then it can be defined as follows [30],


$$\sigma \left(x\right)=\text{max}\left(0,x\right)$$ (2)

There are four FFN blocks interconnected by skip connections in the architecture, to further improve learning and model performance. After the first FFN block processes the input features, its output is fed into the second FFN block. A skip connection connects the output of the first FFN block directly to the input of the second FFN block [31]. This addition helps preserve the original information and gradients, ensuring they flow effectively through the network without vanishing or exploding, which is a common issue in deep learning models [32]. Following this, the output of the combined first and second FFN blocks is passed into the third FFN block. Again, a skip connection adds the output of the combined first and second FFN blocks to the input of the third FFN block. This pattern is repeated with the fourth FFN block as well. These skip connections are crucial for maintaining the integrity of the learned features and gradients across multiple layers, facilitating deeper networks that can learn more complex patterns.

In total, there are three skip connections in the architecture, each strategically placed to enhance the learning process by allowing the model to bypass certain layers, thus maintaining gradient flow, and improving the overall performance of the GNN model.

### 6.2 GNN model

Our proposed GNN model aims to capture intricate patterns and dependencies within the graph structure. The details of the proposed GNN model, including its input representation, message passing, Node embedding, and graph formulation, are presented as follows.

#### Input representation.

The input graph $G=\left(V,E\right),$ where V is the set of vertices or nodes and E is the set of edges, is represented by a tuple consisting of node features, edges, and edge weights. The node features matrix F $\in R^{d\times \left|n_{v}\right|}$ captures the attributes of each node, where d is the dimensionality of the features, and $n_{v}$ is the number of nodes. Each node *μ* is initialized with an embedding $F_{\mu }^{0}=x_{\mu },$ which serves as the starting point for the message passing process. The edges and their associated weights define the relationships and interaction strengths between nodes, providing a structured representation of the data.

#### Message passing layer.

The core of the GNN model is the message passing layer, which iteratively updates node embeddings by aggregating information from neighboring nodes. This iterative process is crucial for capturing the dependencies and interactions within the graph. At each iteration *i*, the embedding of a node *μ* is updated by combining its previous embedding with the aggregated messages from its neighbors [29]:


$$F_{\mu }^{\left(i+1\right)}=Update^{i}\left(F_{\mu }^{i},AGGREGATE\left(i\right)\left(\left\{F_{\mu }^{\left(i\right)},\forall_{\mu }\in N\left(\mu \right)\right\}\right)\right)$$ (3)

$$=Update^{i}\left(F_{\mu }^{i},m_{N\left(\mu \right)}^{\left(i\right)}\right)$$ (4)

Here, $AGGREGATE\left(i\right)$ collects the embeddings from the neighbors of node *μ* to generate a message $m_{N\left(\mu \right)}^{\left(i\right)}$, which is then combined with the previous embedding $F_{\mu }^{i}$ using the $Update^{i}$ function to produce the new embedding $F_{\mu }^{\left(i+1\right)}$. This framework ensures that each node’s embedding captures both its own features and the structural information from its local neighborhood.

#### Node embedding and graph-level formulation.

After $i^{th}$ iterations of message passing, the final node embeddings $Z_{\mu }=x_{\mu },\forall \mu \in V$ encapsulate the aggregated information from each node’s neighborhood. These embeddings can be utilized for various downstream tasks, such as node classification or link prediction. For graph-level tasks, such as graph classification, a pooling operation is applied to obtain a graph-level representation.

The overall graph-level formulation of our model is given by [33]:


$$F^{i}=\sigma \left(AF^{\left(i-1\right)}W_{neighbour}^{i}+F^{\left(i\right)}W_{self}^{\left(i\right)}\right)$$ (5)

Here, A is the adjacency matrix that defines the graph structure, $W_{neighbour}^{i}$ and $W_{self}^{\left(i\right)}$ are learnable weight matrices for the neighboring and self-contributions, respectively, and *σ* is the activation function. This formulation highlights the efficiency of implementing GNNs using sparse matrix operations, enabling the model to handle large and sparse graphs effectively.

### 6.3 Proposed GNN model architecture

The proposed GNN model integrates several components to maximize its predictive capabilities. Initially, the node features are processed through a series of graph convolutional network (GCN) layers. Each GCN layer consists of aggregation functions, combination functions, an activation function, recurrent activation, hidden units, and dropout. The aggregation function collects information from the neighboring nodes, while the combination function integrates this information with the node’s current state. The activation function introduces non-linearity, allowing the model to learn complex patterns.

In our proposed model, we have two GNN layers, each designed to progressively refine the node embeddings. The GCN layer updates the node embeddings and passes the messages to the next GCN layer. This hierarchical approach allows the model to capture both local and global structures within the graph. After the GNN layers, the aggregated node embeddings are passed through the FFN block to make the final prediction.

**Fig 4:**
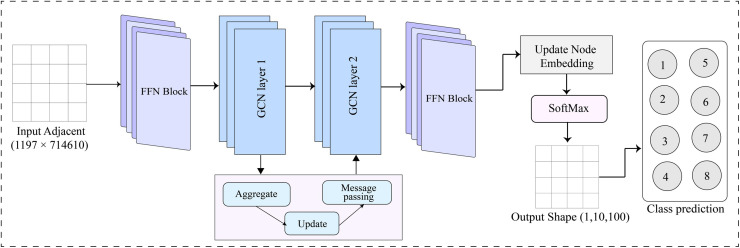
Proposed GNN architecture.

For optimization, we fine-tune the hyperparameters. The hidden units are increased to [32], the learning rate is adjusted to 0.01, and the batch size is increased to 64. A dropout rate of 0.3 is applied to prevent overfitting. We employ the SGD optimizer and activation function ELu. The ConvLSTM1D combination function is employed to effectively process temporal information in sequential data, further enhancing the model’s ability to capture complex dependencies. Fig 4 illustrates the proposed GNN model architecture.

## 7 Result

The efficacy of our GNN model in identifying eight post-anesthesia states are investigated in the results section. We assess the accuracy and robustness of the model, emphasizing the advantages of using SMOTE for addressing these classes which have low data. In order to determine the ideal thresholds, the effect of various threshold settings on the classification accuracy of the GNN is also evaluated. In order to further illustrate GNN’s better capabilities, we also compare its performance with that of ensemble approaches and conventional machine learning models. Our results highlight the GNN model’s efficacy in precisely identifying and distinguishing between post-anesthesia phases, which presents a substantial opportunity for improved patient monitoring and care.

### 7.1 Ablation study

Eight tests are conducted in order to enhance the proposed GNN model’s performance, and the best configuration is chosen for each experiment based on the overall performance. The outcomes of eight ablation study cases are listed in Table 3.

**Table 3:** The results of ablation study.

Convolutional layers			
No.	No. of convolutional layers	Test accuracy (%)	Average Time per step (millisecond)
1	1	84.14	126ms
**2**	**2**	**84.22**	**132ms**
3	3	83.92	128ms
4	4	83.73	140ms
5	5	83.67	150ms
Hidden units			
No.	Hidden units	Test accuracy (%)	Average Time per step
**1**	**64,64**	**86.41**	**128ms**
2	32,32	83.92	8s
Combination type			
No.	Combination type	Test accuracy (%)	Average Time per step
1	concat	83.92	128ms
**2**	**ConvLSTM1D**	**86.99**	**128ms**
Activation function			
No.	Activation function	Test accuracy (%)	Average Time per step
**1**	**elu**	**87.55**	**128**
2	relu	81.44	128
3	tanh	82.11	128
4	softmax	84.44	128
Optimizers			
No.	Optimizer	Test accuracy (%)	Average Time per step
1	Adam	84.22	128ms
2	Nadam	87.55	128ms
3	Adamax	80.91	128ms
**4**	**SGD**	**88.01**	**128ms**
Learning rates			
No.	Learning Rate	Test accuracy (%)	Average Time per step
**1**	**0.01**	**89.66**	**128ms**
2	0.001	88.01	20ms
3	0.0001	85.77	20ms
4	0.0007	65.78	6s
Dropout value			
No.	Dropout	Test accuracy (%)	Average Time per step
1	0.2	89.66	128ms
**2**	**0.3**	**91.01**	**128ms**
3	0.4	84.1	72ms
4	0.5	78.59	20ms
5	0.6	77.89	12ms
6	0.7	74.23	8ms
Batch size			
No.	Batch size	Test accuracy (%)	Average Time per step
1	32	82.87	74ms
**2**	**64**	**92.80**	**7s**
3	128	88.11	128ms

Eight ablation experiments were conducted to improve the performance of the GNN model; test accuracy and average duration per step are highlighted in Table 3. The initial study examined the impact of varying the number of convolutional layers, ranging from 1 to 5. This study revealed that increasing the number of layers above two led to a decline in accuracy, indicating that excessive feature extraction might introduce redundancy. Therefore, two convolutional layers produced the maximum accuracy of 84.22%. In the second study, the evaluation of hidden units showed that a configuration of 64 hidden units achieved a higher accuracy of 86.41% compared to 32 hidden units, highlighting the importance of adequately sized feature representations. The next study was done to find the optimal combination type analysis, where the ConvLSTM1D outperformed simple concatenation with an accuracy of 86.99%, suggesting that temporal dependencies were beneficial for feature extraction. Among the four tested activations, ELU achieved the highest accuracy of 87.55%, outperforming ReLU, Tanh, and Softmax with accuracies of 81.44%, 82.11%, and 84.44%, respectively. While all activation functions maintained the same computational cost of 128ms per step, this experiment emphasizes that selecting an appropriate activation function is crucial for maximizing accuracy. Among optimizers, SGD’s accuracy was 88.01%, demonstrating its effectiveness in training deep networks when properly tuned. Nadam, on the other hand, followed closely with 87.55% accuracy. Adam performed moderately with an accuracy of 84.22%, while Adamax had the lowest accuracy of 80.91%. The learning rate significantly influenced both model performance and training stability. The best learning rate, with an accuracy of 89.66%, was 0.01, indicating that a relatively larger learning rate helped the model converge effectively. However, reducing the learning rate to 0.001 resulted in slightly lower accuracy (88.01%), while 0.0001 further decreased it to 85.77%. A drastic drop was observed when the learning rate was 0.0007, leading to 65.78% accuracy, likely due to slow convergence or getting stuck in local minima. Dropout was used to prevent overfitting, and different dropout values were examined. A dropout of 0.3 achieved the highest accuracy of 91.01%, contributing to the right balance between regularization and information retention. A lower dropout of 0.2 also performed well with 89.66% accuracy. However, increasing the dropout beyond 0.4 led to a significant decline in accuracy, showing degraded performance due to excessive information loss. Additionally, higher dropout rates correlated with lower computational costs, as dropout reduces the number of active neurons during training. 0.7 dropout had the lowest cost of 8ms per step. Finally, a batch size of 64 produced the highest accuracy of 92.80%, highlighting the effect of batch size on model performance, where both smaller and larger sizes resulted in reduced accuracy. The best hyperparameters to maximize the model’s performance are found through this approach. The hyperparameters selected for the GNN model after the experiments are listed in Table 4.

**Table 4:** Hyperparameter’s configuration of the proposed model.

Hyperparameter	Configuration
Activation Function	Elu
Pooling layer	Max-pooling
Epochs	400
Learning rate	0.01
Dropout rate	0.3
Combination type	Concat
Aggregation type	Max
Recurrent Activation	SoftPlus
Hidden Units	[32, 32]
Batch size	64
Optimizer	SGD

The model makes use of a max-pooling layer and the elu activation function. To avoid overfitting, it is trained for 400 epochs using a learning rate of 0.01 and a dropout rate of 0.3. Concatenation (Concat) is used for feature combining, while max aggregation is used for node information. SoftPlus is the recurrent activation function; it has two hidden layers, each having 32 units. For training, the batch size is set to 64.

With these hyperparameter settings, the model achieves the optimal performance. Table 5 presents the test accuracy and Kappa statistics of the proposed model.

**Table 5:** Proposed Model performance.

Model Performance attributes	Result
Model Name	GNN
Test Accuracy	92.80%
Cohen’s Kappa statistic	92.00%
Epoch	400
Amount of graph data	1297×714610

The performance statistics of the proposed GNN model are shown in Table 5. With a test accuracy of 92.80%, the model demonstrated a high degree of forecast precision. The model’s reliability is further supported by the Cohen’s Kappa statistic, which shows a 92.00% agreement between the model’s predictions and actual results. With more than 400 training iterations, the model was thoroughly optimized and learned.

### 7.2 Performance analysis of the proposed GNN model

**Fig 5:**
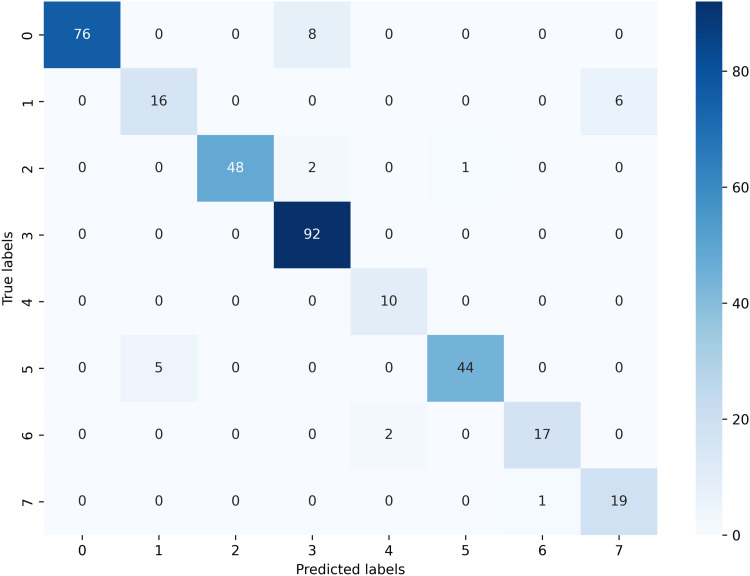
Confusion metrics of the proposed GNN model.

The set of performance indicators is used to systematically evaluate the effectiveness of the proposed model. This evaluation includes metrics such as Matthew’s correlation coefficient (MCC), false positive rate (FPR), false discovery rate (FDR), and false negative rate (FNR). Other indicators include test accuracy, recall, precision, training accuracy, and F1 score. These metrics are computed using the confusion matrix’s true positives (TP), true negatives (TN), false positives (FP), and false negatives (FN) values. Fig 5 shows the confusion matrix for the combined dataset’s test set.

From this confusion matrix, several evaluation metrics can be analyzed to evaluate the model performance [34]. Table 6 represents the evaluation metrics of the GNN model.

**Table 6:** Evaluation metrics of the proposed model.

Performance metrics	Result (%)	Performance metrics	Result (%)
Test Accuracy	92.8	NPV	99.31
Sensitivity	92.64	FPR	0.47
Precision	92.71	FDR	9.3
Specificity	99.30	FNR	7.36
MCC	90.86	F1 Score	91.39

The evaluation metrics for the proposed GNN model, which show both its overall performance and classification quality, are shown in Table 6. With a test accuracy of 92.8%, the model demonstrated outstanding prediction accuracy. The model’s strong sensitivity (recall) and precision, which stand at 92.64% and 92.71%, respectively, show how well it can recognize true positives and make accurate predictions about pertinent cases. The model’s ability to identify real negatives is demonstrated by its 99.30% specificity, and its reliability is further highlighted by the NPV score pf 99.31%. Few incorrect positives are indicated by the model’s low FPR of 0.47% and FDR of 9.3%. At 7.36%, the FNR indicates a low number of false negatives. A balanced performance across all classes is indicated by the MCC, which is 90.86%. Ultimately, 91.39% is the F1 score—a measure of recall and precision—which validates the model’s strong performance in classification tests.

**Fig 6:**
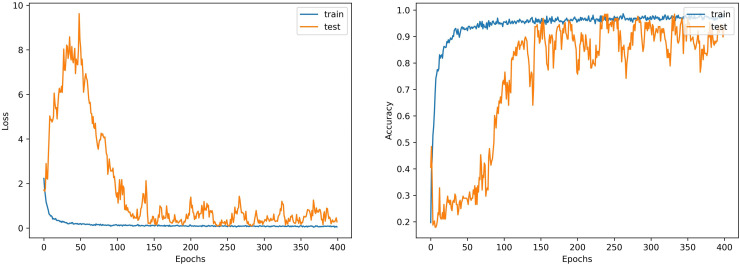
Loss and accuracy curves of GNN model.

The accuracy and loss curves both illustrate the training dynamics and provide insight into the model’s learning process. Effective generalization of the training data is demonstrated by the model’s constant reduction of loss and rise in accuracy. Fig 6 shows the accuracy and loss curves of the model.

The model successfully decreases training loss and achieves high training accuracy, as demonstrated by the accuracy and loss curves, indicating successful learning. At lower numbers, the test loss first declines before stabilizing with occasional swings. In a similar vein, test accuracy increases rapidly and maintains a high level of variability. This indicates that the model has a stable general performance across 400 epochs, retaining low error rates and high accuracy on both training and test datasets.

**Fig 7:**
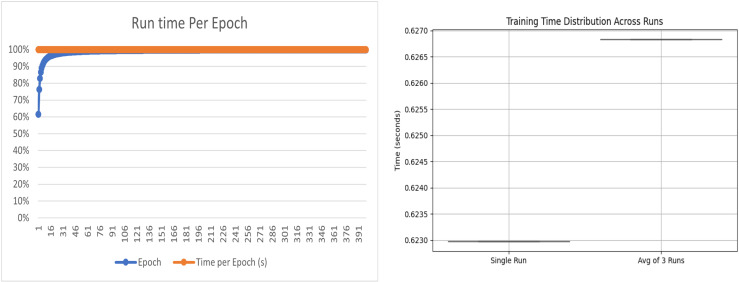
Run time analysis of the proposed model.

Additionally, we conducted a detailed analysis for the runtime evaluation of our proposed model. First, we evaluated the runtime for each epoch individually. Then, we compared these results with the average runtime over three runs. The curves derived from this analysis are presented in Fig 7.

The figure shows two subplots that assess the runtime efficiency of the training process. The left subplot illustrates the runtime per epoch, wherein the blue dots denote the duration of individual epochs, and the orange line reflects the cumulative trend. Initially, the runtime exhibits variability; however, it rapidly stabilizes around 100% efficiency, signifying a consistent computational load. Conversely, the right subplot shows the distribution of training times between a singular run and the average of three consecutive runs through a box plot representation. The constricted range (approximately 0.623s to 0.627s) attests to minimal variance, thereby corroborating both the efficiency and stability of the training process. In summary, the findings reveal a consistent runtime per epoch alongside a resilient computational performance.

### 7.3 GNN model’s performance on different data augmentation methods

In this study, we utilized SMOTE as the data augmentation method, which was introduced by Chawla et al. [35], is a widely adopted technique for addressing class imbalance issues. SMOTE can generate synthetic samples through interpolation between existing minority class instances, rather than duplicating them. However, to demonstrate the effectiveness of SMOTE a performance comparison of the proposed GNN model is shown in Table 7 where additional three augmentation techniques of Angle Radius-Adaptive Synthetic Sampling (ARADASYN), random oversampling and synthetic oversampling methods are employed.

**Table 7:** Performance of GNN model on different augmentation techniques.

Augmentation techniques	Proposed GNN model’s performance
SMOTE	92.8%
ARADASYN	89.78%
Random oversampling	85.33%
Synthetic oversampling	81.67%

Table 7 shows that the performance of the GNN model is highest with the SMOTE augmentation technique. The ARADASYN method, which adapts sample generation based on the angle and radius of data points [36], resulted in a slightly lower performance of 89.78%, indicating its effectiveness but not as much as SMOTE. Random oversampling, which duplicates minority class instances [37], showed a performance of 85.33%, while synthetic oversampling, generating entirely new synthetic samples [38], yielded the lowest performance at 81.67%, likely because these samples did not fully capture the true data distribution. Unlike random oversampling, which risks overfitting by replicating identical samples, synthetic oversampling techniques reduce the risk by generating new data points. However, the risk of overfitting is not entirely eliminated. SMOTE creates more diverse and informative synthetic samples, ensuring a well-balanced dataset [39]. This approach maintains the underlying structure of the minority class, improving feature representation and making it particularly effective for small and imbalanced datasets. By leveraging SMOTE, the GNN model can learn more generalized patterns, which reduces bias towards the majority class and enhances the model’s overall classification performance. This results in better generalization and robustness, allowing the model to effectively classify the wakefulness and deep sleep states in post-anesthesia conditions.

### 7.4 Effectiveness of SMOTE in GNN model

We used SMOTE in our study to account for less amount of data in the dataset, which improved the accuracy of the model. SMOTE enhanced the model’s capacity to learn from a more balanced distribution of data by creating synthetic samples for the underrepresented classes. The implementation of this strategy yielded notable improvements in classification accuracy and overall model performance. The performance of the model before and after applying SMOTE is analyzed in Table 8.

**Table 8:** Performance of the model before and after applying SMOTE.

Performance Metrics	Before SMOTE	After SMOTE
Test accuracy	84.21	92.8
Sensitivity	40	92.64
Precision	43.47	92.71
Specificity	92.64	99.30
NPV	91.46	99.31
FPR	18.93	0.47
FDR	61.95	9.3
FNR	64.92	7.36
F1 Score	39.39	91.39
MCC	16.43	90.86

**Fig 8:**
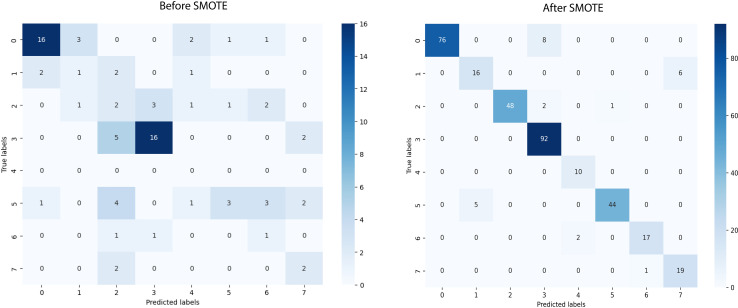
Confusion matrix of before SMOTE and after SMOTE.

The confusion matrix of before and after applying SMOTE is illustrated in Fig 8.

### 7.5 Evaluating GNN model’s performance on different edge weight calculation methods

In this section, we compare the impact of different edge weight calculation methods including, Spearman correlation, Cosine similarity [40], and Euclidean distance [41], on the performance of the proposed GNN model. Table 9 presents the results for each method in terms of classification accuracy.

**Table 9:** Performance Comparison of Edge Weight Calculation Methods in GNN Model.

Edge Weight Calculation Method	Accuracy (%)
Spearman Correlation	92.8
Cosine Similarity	85.57
Euclidean Distance	89.67

Table 9 shows that Spearman correlation outperforms both Cosine similarity and Euclidean distance, achieving the highest accuracy of 92.8%. It is evident that, cosine similarity, with an accuracy of 85.57%, and Euclidean distance, with an accuracy of 89.67%, are less effective in the classification task with GNN. Though cosine similarity may be helpful for high-dimensional data, it primarily measures the angle between vectors. It ignores the magnitude, which limits its ability to identify nuanced relationships in the data. On the other hand, Euclidean distance measures the straight-line distance between points, does not consider the rank order of the data, and may be influenced more by outliers or extreme values, leading to a lower accuracy compared to Spearman correlation. Therefore, both methods fail to capture the essential monotonic relationships between features as effectively as Spearman correlation, resulting in reduced model performance. Thus, Spearman correlation’s ability to prioritize the relative ranking of features allows the GNN model to learn more meaningful representations, improving generalization and enhancing classification accuracy.

### 7.6 Evaluating different threshold value of Spearman correlation

The performance of the model is compared with different threshold levels in this experiment. This experiment helps determine the ideal threshold value for the graph creation, preserves significant features, and improves accuracy. Table 10 presents the findings.

**Table 10:** Results of the GNN Model performance on different threshold value according to Spearman correlation.

Threshold	Test Accuracy (%)	Total edge number
<= 0.7	64.23	1191
<= 0.8	68.14	1194
<= 0.9	73.91	2374
<= 0.93	75.41	2385
<= 0.97	81.29	4696
**<= 1**	**92.80**	**714610**

Table 10 illustrates the way various threshold configurations affect the GNN model’s performance as determined by Spearman correlation-based test accuracy and total edge count. The model’s test accuracy steadily improves as the threshold value rises. The model attains a test accuracy of 64.23% with 1,191 edges at a threshold of <= 0.7. With a minor rise in edge numbers, the accuracy increases to 68.14% and 73.91%, respectively, when the threshold is gradually raised to <= 0.8 and <= 0.9. The accuracy of the model with 2,385 edges achieves 75.41% with a threshold of <= 0.93. When the accuracy reaches a threshold of less than 0.97, it increases significantly to 81.29% with 4,696 edges. Ultimately, the model attains its maximum accuracy of 92.80% with a substantially greater total edge number of 714,610 at the maximum threshold of <= 1. This graph shows that by giving deeper connectivity information, adding more edges—up to the maximum threshold—improves the accuracy of the model.

### 7.7 Comparison with 1D CNN models

In this section, the proposed GNN model’s performance is evaluated against CNN. In the context of the classification, we seek to illustrate the benefits and drawbacks of each technique by comparing important performance measures among different models. This comparative analysis sheds light on how well the GNN model performs in terms of reaching high accuracy and dependability when compared to conventional approaches. Table 11 presents the comparison table with 1D CNN model.

**Table 11:** Comparison table with 1D CNN models.

Performance Metrics	1D CNN	GNN
Test accuracy	70.41	92.8
Sensitivity	56.62	92.64
Precision	70.20	92.71
Specificity	70.41	99.30
NPV	97.12	99.31
FPR	29.58	0.47
FDR	29.79	9.3
FNR	23.37	7.36
F1 Score	68.11	91.39
MCC	63.87	90.86

A GNN and a 1D convolutional neural network (1D CNN) are compared in Table 11. In every parameter, the GNN performs better than the 1D CNN: it has a higher test accuracy (92.8 vs. 70.41), indicating that it produces more accurate predictions. Additionally, the GNN has improved precision (92.71 vs. 70.20) and sensitivity (92.64 vs. 56.62), indicating that it is more adept at correctly recognizing positive cases and producing positive predictions. Furthermore, the GNN shows improved negative predictive value (99.31 vs. 97.12) and specificity (99.30 vs. 70.41), indicating its capacity to recognize negative situations with accuracy. Less false positives (0.47 vs. 29.58), false negatives (7.36 vs. 23.37), and false discoveries (9.3 vs. 29.79) were observed. The F1 score (91.39 versus 68.11) and Matthews Correlation Coefficient (90.86 versus 63.87) of the GNN, when compared to the 1D CNN, further validate its advantage in terms of effectiveness and dependability in the classification tasks.

### 7.8 Experiments with different dataset

To evaluate the efficiency of our GNN model on a broader range of data, we expanded our experimental approach by incorporating additional datasets. Specifically, we utilized six datasets from the Zenodo repository (https://zenodo.org/records/8348272), all in Version 3 format, which include diverse anesthesia states. These datasets were combined with our primary dataset, resulting in a comprehensive merged dataset of 872 samples representing nine distinct anesthesia states, including two previously unrecorded states: Eyes Open (EO) and Eyes Closed (EC). A detailed description of the datasets is provided in Table 12.

**Table 12:** Dataset description.

Dataset	Brain States									
	W	DS	EO	EC	N3	UWS	REM	N2	BI	HC
WvsDS_GScoactivatin	30	23								
WvsDS_random_point	30	23								
EOvsEC_GScoactivation			67	67						
EOvsEC_random_point			67	67						
N3vsUWS_GScoactivation					10	36				
N3vsUWS_random_point					18	36				
REMvsWvsN3_GScoactivation	19				10		7			
REMvsWvsN3_random_point	19				10		7			
WvsN2vsN3_GScoactivation	30				18			37		
WvsN2vsN3_random_point	30				18			37		
BIvsUWS_GScoactivation	15					19				
BIvsUWS_random_point	15					19				
HCvsUWS_GScoactivation						19				21
HCvsUWS_random_point						19			8	21
Subtotal without SMOTE	188	46	134	134	84	148	14	74	8	42
Subtotal with SMOTE	217	63	184	184	126	203	19	101	41	57

The table provides a comprehensive summary of datasets utilized for the classification of nine anesthesia states, which include Wakefulness (W), Eyes Open (EO), and Unresponsive Wakefulness Syndrome (UWS). The data are derived from GScoactivation and random point techniques, encompassing a total of 872 samples. This varied compilation facilitates advanced model training and enables a more profound analysis of the differentiation of brain states. Further, we slightly augmented the dataset, and we got 1195 samples with nine classes.

**Fig 9:**
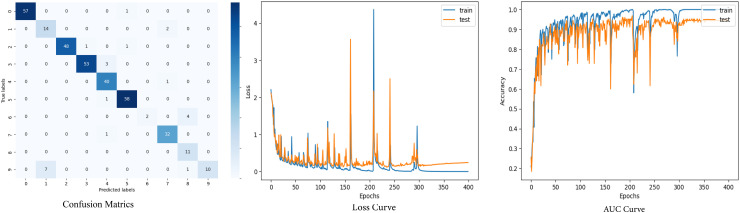
Confusion matrices, ROC curve, and AUC curve of the merged dataset.

In order to assess the efficacy of the GNN framework on the augmented dataset, a confusion matrix and ROC and AUC curves were constructed. The confusion matrix offers a comprehensive analysis of the classification outcomes, emphasizing the model’s proficiency in accurately distinguishing among the nine anesthesia states. The confusion metrics and Curves are shown in Fig 9.

The evaluation metrics several performance metrics are analyzed. These evaluation metrics are shown in Table 13.

**Table 13:** Evaluation metrics of the merged dataset derived from the confusion metrics.

Performance metrics	Result (%)	Performance metrics	Result (%)
Test Accuracy	93.39	NPV	99.23
Sensitivity	91.31	FPR	0.41
Precision	93.82	FDR	9.91
Specificity	97.41	FNR	6.84
MCC	91.82	F1 Score	92.34

The table presents the performance metrics of a classification model, showcasing strong results across various measures. The model achieves a high-test accuracy of 93.39%, with sensitivity (91.31%) and precision (93.82%) indicating that it correctly identifies positive cases and minimizes false positives. Specificity (97.41%) further demonstrates the model’s ability to accurately detect negative cases. The MCC (91.82%) reflects a well-balanced performance overall. Additionally, the model boasts an impressive NPV of 99.23%, indicating reliable negative predictions, and a low FPR (0.41%) and FDR (9.91%), signifying few incorrect positive classifications. The FNR (6.84%) remains relatively low, showing that the model rarely misses positive cases. The F1 Score (92.34%) further underscores the model’s balanced performance between precision and recall, making it a highly effective classifier.

These metrics demonstrate the significant performance of the GNN model built for our proposed approach. Although we observed an improvement in performance on this new dataset by almost 1%, it is important to note that the dataset we used for this study’s classification task, combines eight distinct datasets, resulting in a highly imbalanced and yet relatively small-sized dataset. Compared to this dataset, our original dataset is significantly smaller but contained greater variation. Despite this, the model achieved a commendable accuracy of 92.8%, showcasing the effectiveness of the GNN model. Therefore, although the additional dataset demonstrated good performance, our method provides a more robust solution and superior experimental results.

**Fig 10:**
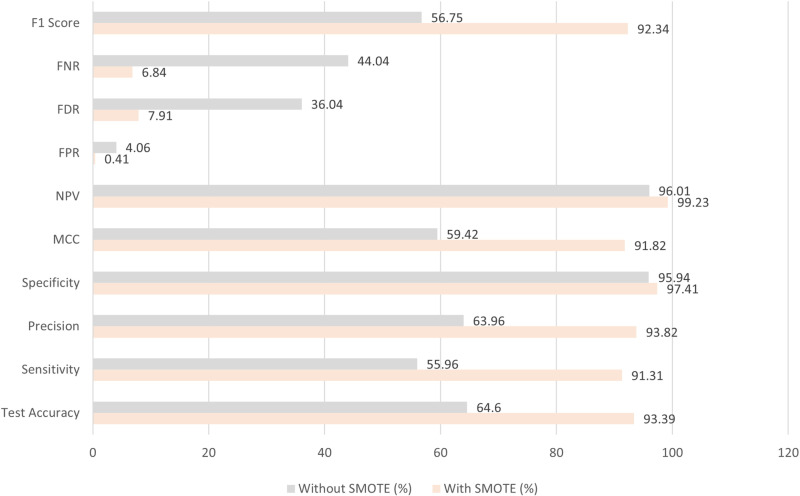
Performance comparison with SMOTE and without SMOTE in merged dataset.

Furthermore, we executed experimental analyses on this dataset utilizing the GNN framework, both inclusive and exclusive of the SMOTE. The findings indicated that, in the absence of SMOTE, the performance of the proposed models was markedly inferior. A comparative assessment of the evaluation metrics, with and without the application of SMOTE, is shown in Fig 10, underscoring the beneficial effect of SMOTE on model efficacy.

### 7.9 Comparison with previous studies

**Fig 11:**
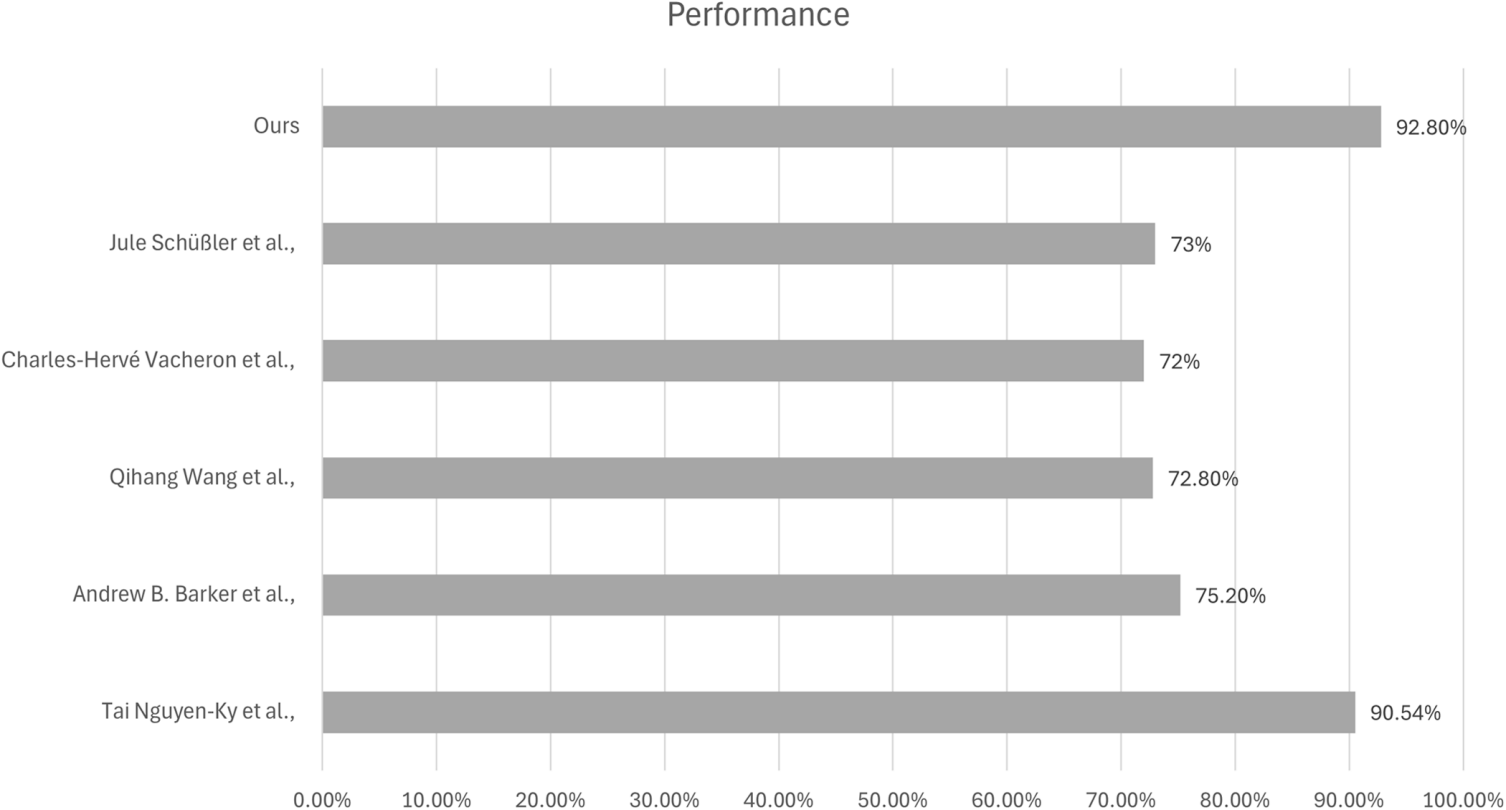
Performance comparison with previous studies.

Further, a comparative analysis with previous studies is conducted for a robust analysis of the performance of our GNN model. Fig 11 illustrates the performance comparison with previous studies.

With a test accuracy of 92.80%, our GNN model performs significantly better than the models used in previous research, demonstrating its greater capacity to identify post-anesthesia conditions. In contrast, a high accuracy of 90.54% was attained by Tai Nguyen-Ky et al.‘s [9] technique, which processes EEG signals using the Hurst method. Though still amazing, the increased accuracy of our GNN model suggests more efficient feature extraction and classification capabilities. In their study, Andrew B. Barker et al. [42] used machine learning models to predict unexpected care escalations (UCE), and they achieved a noteworthy accuracy of 75.20%. Although their method successfully identified important patient risk variables, it is not as accurate as our GNN model. This suggests that GNNs may be able to handle intricate temporal dependencies and feature interactions better than traditional machine learning techniques. Anes-MetaNet, a deep learning system with 72.80% accuracy that combines CNNs and LSTMs, was proposed by Qihang Wang et al. [22] Even with the application of sophisticated deep learning algorithms, the performance is still significantly lower than our GNN model, suggesting that our method may be more appropriate for managing the noise and variability present in the EEG data used to classify anesthetic states. Notable works were also given by Jule Schüßler et al. [43] and Charles-Hervé Vacheron et al. [44], with accuracies of 72% and 73%, respectively. While Schüßler’s research sought to identify delirium in the PACU through preoperative EEG recordings, Vacheron’s work employed a classification tree analysis to examine task interruptions in a PACU. Although both studies are useful, they show inferior accuracy when compared to our GNN model. Overall, our GNN model outperforms previous approaches in the literature in identifying post-anesthesia states, as evidenced by its superior accuracy of 92.80%, which highlights its robustness and effectiveness. This demonstrates how GNNs can improve the accuracy and consistency of anesthetic state identification in clinical situations.

### 7.10 Practical implications and clinical integration of the proposed classification model

The integration of advanced machine learning models into anesthesia monitoring systems has the potential to transform the way anesthesia depth is assessed and managed, making it a critical area of development. Traditional monitoring systems primarily rely on indirect physiological metrics such as heart rate, blood pressure, and respiratory rate to estimate the depth of anesthesia [45]. While these measures are useful, they do not provide a direct or accurate representation of the patient’s state of consciousness. In many cases, these physiological indicators can be influenced by multiple factors such as individual variability, medication effects, and patient conditions, leading to inconsistencies in monitoring. Furthermore, these metrics often fail to capture the subtle transitions between different states of consciousness, especially those induced by anesthesia [46,47]. The limitations of these conventional methods underscore the importance of more accurate, brain-specific monitoring approaches that can provide real-time, direct feedback on the patient’s level of consciousness. This study introduces a novel approach by leveraging a GNN model to analyze numerical brain activity data and classify anesthesia-induced transitions between wakefulness and deep sleep states. The advantage of using a GNN lies in its ability to model the complex, non-linear relationships between different brain regions represented as numerical features. These numerical data points are crucial in understanding the nuanced levels of consciousness during anesthesia. Unlike traditional methods that rely on simplistic measures or manual interpretations, the GNN model can process and interpret these intricate interdependencies, providing detailed, and continuous measure of anesthesia depth.

Our proposed graph-based approach using GNN offers superior decision-making and interpretability compared to traditional machine learning models and 1D CNN for numeric dataset analysis. Unlike conventional machine learning models that process feature vectors independently, by examining the graph structure formed from the numerical data, where each node represents a state or feature and edges capture the correlation between them, the GNN approach offers a deeper understanding of the relationships within brain activity [48,49]. Similarly, while 1D CNNs are effective in learning spatial dependencies within sequential data, they do not explicitly model relationships between data samples, potentially missing critical correlations in small and imbalanced datasets. The ability to analyze how different node features influence the classification process based on the strong connection between the similar nodes, makes the model more reliable in decision making. As the nodes with similar classes tends to build strong connection with each other within the graph dataset, it makes the GNN model easier to classify the nodes even if the dataset is small and imbalanced [29]. This method moves beyond isolated features and accounts for the broader, interconnected network of relationships that dictate the transitions in the brain’s state. To assess the performance of the proposed GNN model, we compared it with 1D CNN and six widely used machine learning models (See Table 14) including, Support Vector Machine (SVM), Random Forest, Decision Tree, Logistic Regression, K-Nearest Neighbors (KNN), and Naive Bayes.

**Table 14:** Performance comparison between proposed model and traditional techniques.

Classification models	Accuracy (%)
Proposed GNN model	92.8
SVM	84.55
Random Forest	86.32
Decision Tree	80.29
Logistic Regression	78.61
KNN	75.44
Naive Bayes	71.91
1D CNN	70.41

The results, summarized in Table 14, reveal a clear performance advantage of the GNN model. As shown in the table, the GNN achieved an accuracy of 92.8%, significantly outperforming all traditional machine learning models. The SVM, which is widely used for classification tasks, reached an accuracy of 84.55%, while Random Forest scored 86.32%. Other models, including Decision Tree (80.29%), Logistic Regression (78.61%), KNN (75.44%), Naive Bayes (71.91%) and 1D CNN (70.41%), demonstrated even lower accuracies. This stark contrast highlights the limitations of traditional methods when dealing with complex numerical data, especially in the context of brain activity, where subtle relationships between features are crucial for accurate classification. In conclusion, integrating this GNN-based classification model into anesthesia monitoring systems offers a transformative shift toward precision medicine. It directly addresses the brain’s state of consciousness, overcoming the limitations of traditional methods by providing more reliable and timely feedback for anesthesiologists. Additionally, the ability of the GNN model to manage imbalanced data sets, optimize drug dosages, and enhance intraoperative and postoperative care is significant for improving patient safety and clinical outcomes. This approach marks a significant advancement in anesthesia monitoring and can substantially impact anesthesiology practices and patient care in the future, establishing a pivotal role in clinical decision-making.

## 8 Discussion

This study used a proposed GNN model that achieves 92.80% accuracy and performed better than other state-of-the-art approaches. One of the key reasons might be the GNN model identifies all connections between the features taking them as a graphical representation. In this graphical representation, the strong node-edge relationship played a vital role which is identified using a well-known approach named Spearman correlation. Notably, through an ablation study, the design of the model is optimized and its ability to adapt is increased. Additionally, the dataset was quite challenging with less amount of data, and thus to address this, SMOTE is applied and came up with an optimal performance. In summary, our GNN model not only outperforms current techniques but also presents a novel approach for identifying feature connections in post-anesthesia state analysis. This model has the potential for useful application in the medical field, which could ultimately improve patient outcomes, with additional validation and dataset expansion. One key concern regarding the practical applicability of the model is its evaluation on real-world patient data. Clinical datasets often exhibit greater complexity due to variations in patient demographics, anesthesia protocols, and physiological responses. In this study, we utilized publicly available datasets, which, although diverse, may not fully capture the variability present in real-world clinical settings. The lack of access to private patient data limited our ability to directly validate the model in a clinical environment. However, to ensure a broad data distribution, we merged seven datasets for classification analysis. Additionally, we tested our model on six more datasets from Zenodo and observed no significant performance drop. This demonstrates the reliability of the proposed GNN model, further enhancing its robustness and applicability. However, in future study, our aim is to incorporate private dataset having real-world patient data to further demonstrate the robustness of the proposed approach. The ability of the GNN model to generalize across multiple datasets suggests its potential to change surgical processes by giving medical professionals a trustworthy tool to track patients’ post-anesthesia states. Subsequent research endeavors might be centered on expanding the dataset and implementing the model in practical scenarios to strengthen its efficacy and amplify its applicability.

## 9 Conclusion

Accurate classification of post-anesthesia conditions is essential to ensure the safety of patients and enhance recovery procedures in medical environments. Flawed classification may result in insufficient patient supervision, which may cause problems or postpone interventions. In this work, we have used machine learning algorithms on a merged dataset of eight classes with the goal of increasing the classification accuracy of post-anesthesia conditions. A combined dataset of 325 patients was obtained by merging seven datasets. The SMOTE technique is used to increase the size of the dataset to 1196 samples. We built a graph and proposed a GNN model to carry out the classification task. By using an ablation study to assess several hyperparameters, we were able to obtain a 92.80% test accuracy. The hyperparameter setup of the GNN model, performance analysis of the GNN, the usefulness of SMOTE in improving the GNN model, the influence of various Spearman correlation threshold values, and a comparison with a 1D CNN were conducted for a rigorous evaluation of the proposed approach. When compared to the 1D CNN, the GNN model showed notable gains in sensitivity, precision, and overall accuracy, among other performance criteria. The performance of the GNN was improved by the implementation of SMOTE. Furthermore, analyzing various spearman correlation criteria provides valuable information about how to choose best features optimally for an improved model performance. In conclusion, our research shows that GNN models may effectively classify post-anesthesia conditions, particularly when paired with methods such as SMOTE to rectify data scarcity issue. The performance of the proposed GNN model highlights its potential usefulness in anesthesiology clinical decision-making.
